# Discussion on Mr. Nelson Hardy's Paper

**Published:** 1889-02-02

**Authors:** 


### Contributions by Patients to Hospitals.

### DISCUSSION ON MR. NELSON HARDY'S PAPER.

At a meeting of the members of The Hospitals Association, held on Wednesday, January 23rd, at St. Mary's Hospital, under the presidency of Sir Edward Sieveking, Mr. H. Nelson Hardy read the paper an abstract of which was published last week.

The Chairman expressed regret at the unavoidable absence of the President of the Association, Dr. Bristowe, and of Mr. Burdett-Coutts, M.P., who had previously brought the subject of patients' payments before the Association. There were, no doubt, many faults to be corrected in our hospital system, but the defects in connexion with the treatment of patients and payment by them were as old as he was, and he remembered a general practitioner telling him fifty years before that his practice had been ruined by the out-patients' department of the hospital in the town where he lived. The Chairman concluded by proposing a vote of thanks to Mr. Hardy, which was carried with acclamation.

A Gentleman in the meeting suggested that, while the remedy for existing evils was not very clear, some of them might be met by every patient who went to a hospital being required to give a certificate of the amount of the income-tax he paid. The payment required of him by the hospital should be based upon that amount. The difficulty had been met in this way in India.

Mr. George Brown, M.R.C.S., in a long speech, contended that this question of patients' contributions to hospitals was more a consultants' than a general practitioners' question, and drew a deplorable picture of the present position of many consultants in London, the result, he alleged, of indiscriminate relief being given by the large general hospitals. Most respectable families sent their sick children or friends to a hospital instead of having a consultant, and the medical man was helpless. As a remedy, he suggested that each hospital physician and surgeon should be allowed to do what he said not one dared to do at the present time, put certain questions to the patients, and refuse to treat them if he thought they ought not to receive charity. He spoke strongly on the way in which special hospitals were established, and maintained that charity and a pay system would not work together in hospitals.

Mr. Timothy Holmes deeply regretted the absence of Mr Burdett-Coutts, M.P., because he considered that Mr. Hardy had hit upon a very great blot in that gentleman's paper, which was that Mr. Burdett Coutts did not touch on the important, and he considered vital question of how his proposal to make the patients of the general hospitals pay, affected the interests of medical education. Without the discussion of that, he maintained that the whole thing was *en l'air*. The general hospitals of London now occupied a different relation towards the poor to that which they held in the times when there were no poor-law medical institutions of any kind. The suggested disinclination of the poor to enter poor-law hospitals and poor-law out-patients departments, rested upon no foundation of any kind, for the institutions in question were perfectly good, were perfectly sufficient for their purpose, were organised with the utmost care, and conducted with the greatest possible skill and zeal, and entirely relieved the ratepayers of London from all duty whatever in the medical care of the great body of the extremely poor. Therefore those hospital reformers who discussed the question without taking that matter into consideration, neglected the very first and most important consideration in the whole question. The general hospitals were now no longer organised as they used to be, for the benefit of the extremely poor, for paupers were amply and well provided for. The great general hospitals were founded and maintained in a great measure for the promotion of medical education, and for the relief of such maladies as could hardly well be undertaken without special medical education. That, he thought, cut away a good deal of the basis of the institution of special hospitals, and it was perfectly true that the general hospitals had themselves to thank for the establishment of a good many of these institutions. Mr. Burdett-Coutts's singular arithmetical proposition, that by making a smaller loss you could increase your business had puzzled him for some time, but he found it beyond his arithmetical faculties. His impression as to pay hospitals was that there ought to be two different classes of institutions. Large general hospitals, while incidentally treating the poor, were founded for the purpose of carrying on medical education; and while he was favourable to pay hospitals, he argued that they ought to be founded frankly and fairly upon a commercial basis. They were appointed to supply medical and surgical advice, and the persons who supplied it must be the first remunerated. All the systems of pay hospitals left those unfortunate persons out in the cold, or pretty nearly so. It seemed to him one of the worst examples of the sweating system he had ever heard of, to sell medical advice at such a rate as, while it kept up the institution, left next to nothing for the labourer upon whose efforts and labour that institution subsisted. It was utterly unfair and improper; and if the pay hospitals were founded, they should be established in such a way that the medical officer either received a substantial salary, or a fixed proportion of the receipts. He agreed most heartily with the proposition which was made for supplementing the resources of the general hospitals by contributions from the working classes, and those contributions, he thought, might very fairly be organised by The Hospitals Association. He referred to the difficulty of obtaining anything like concerted action between the various hospitals and medical charities of the metropolis, and said no thorough and effectual reform would be obtained in the absence of that concerted action. Moreover, the concerted action would not be obtained in the absence of pressure of public opinion intelligently directed upon the medical charities. Some exhaustive inquiry must be instituted by a competent body responsible to the public. Such an inquiry would crush out of existence many charities which ought never to have been instituted, and which were founded exclusively for private ends. He concluded by moving that the Council of The Hospitals Association should be requested to take into consideration the question of organised workshop contributions in aid of our general hospitals.

Mr. Smith, a medical practitioner, seconded the motion, remarking upon the suggestion of the income tax basis for calculating payments for hospital patients, and expressed the opinion that such a system would never answer. He thought the question of the deficiencies was not so much one of contributions as of a stoppage of the existing abuses. He urged that medical students should be allowed in the poor-law asylums, and that The Hospitals Association should endeavour to obtain a Royal Commission on the matter, in order to bring pressure to bear on the Local Government Board. He considered the Hospital Saturday Fund had done more harm than good, because workpeople paid their small contributions, and considered that exonerated them from everything further, and that they had a perfect right to gratuitous aid.

Dr. Gilbart Smith agreed that the two systems of paying and non-paying patients should be kept distinct, but did not consider that the provident system could ever be carried on, even in the out-patients' department, with a school attached The question had been brought forward that week at the London Hospital by one of its best friends, Sir Edmund Currie, who was most anxious that the provident system should be adopted. The governors, however, came to the conclusion that it would be utterly impossible to work that system with such a hospital as the London. Paupers should be treated at the poor-law institutions, and those patients able to pay would be suitable cases for the provident institutions, but between these two classes came a class who were not paupers, but who were unable to pay anything for hospital treatment. That was a large class, and it was for it that a large general hospital existed. He disagreed, *in toto*, with the remarks of Mr. George Brown, and considered that they were without foundation. For himself, he was always able to say of an unsuitable patient, "No, I will not see that case," and he never saw a case that struck him as not being a fit case for a hospital.

The resolution was put, and carried unanimously.

Mr. Burdett said he ventured to think that Mr. Burdett-Coutts's figures were not at fault, as had been suggested, and the reader of the paper had simply failed to grasp their meaning. Mr. Burdett-Coutts was engaged for a year on his paper, and it was quite worthy of being considered a classical contribution to the discussion of hospital questions. This question was approached from the point of view that the London hospitals had a great number of unoccupied beds, and, moreover, that a large number of the beds occupied were actually occupied by people who could afford to pay something. Therefore, said Mr. Burdett-Coutts, assuming that the contention as to the condition of the patients was correct, and remembering the number of beds unoccupied for want of funds, if a certain number of persons who could pay did pay, that would provide an additional income to the institutions, which would allow them to open more beds. Thus, if the cost of in-patients was 5s. each per day, and five of the existing patients were induced to contribute varying sums, amounting in all to 10s. per diem, that 10s. would represent the cost of maintaining two additional beds. If that money were therefore taken from the patients those two beds could be occupied at the cost of the contributions of the patients, simply by doing what the general practitioners advocated, that is by the establishment of a check upon the present alleged abuse of hospitals. He considered it was only just to Mr. Burdett-Coutts that these figures should be explained, in order that his arguments might be fairly understood, and he could assure the meeting that the figures would bear the closest investigation.

### OUR LIFE.

"O we live, O we live—
And this life that we perceive
Is a strong thing and a grave,
Which for others' use we have,
Duty-laden to remain.
We are helpers, fellow-creatures,
Of the right against the wrong;
We are earnest-hearted teachers
Of the truth which maketh strong."

—*E. B. Browning.*

